# Bringing Evo Devo to Life

**DOI:** 10.1371/journal.pbio.0030340

**Published:** 2005-10-11

**Authors:** Paul M Brakefield

## Abstract

Sean Carroll's book shows how the central black box of development that maps phenotypes onto genotypes is revealing its secrets in many exquisite, and frequently unexpected, ways.

[Fig pbio-0030340-g001]

**Figure pbio-0030340-g001:**
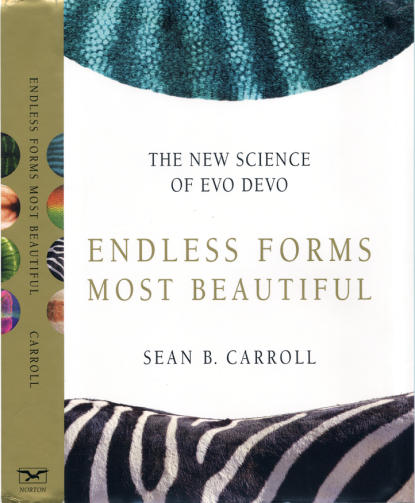
Carroll SB (2005) Endless forms most beautiful: The new science of evo devo and the making of the animal kingdom. New York: W. W. Norton and Company. 350 p. ISBN (hardcover) 0-393-06016-0. US$25.95

The essential path of adaptive evolution is that genetic variation among individuals is translated by the process of development into phenotypic variability, which is then screened by natural selection for any design changes that enhance survival and reproductive success. In *Endless Forms Most Beautiful*, Sean Carroll shows how the central black box of development that maps phenotypes onto genotypes is revealing its secrets in many exquisite, and frequently unexpected, ways. This is at last enabling the whole evolutionary trail, from genes to adaptive phenotypes, to be traced—the promised synthesis begins to be realized. Changes in development generate the variation in morphology on which natural selection can act—no variation, no evolution [1]. To an evolutionary biologist, the exciting potential of evolutionary developmental biology, or “evo devo,” is the ability to open up this black box in an evolutionary context. Opening this black box will reveal not only how developmental processes themselves evolve, but also how these processes can in turn influence the paths and trajectories of morphological evolution.

Any new field in science needs its standard-bearers. In Sean Carroll evo devo has such a champion. His book, together with the new edition of an earlier book that he co-authored, called *From DNA to Diversity* [2], covers the whole research field. Evo devo illustrates as never before how Darwin's “endless forms most beautiful” have been and are still being made. Embryology has always been an integral component of the evidence for evolution and the principle of common descent. In Darwin's time, it was a challenge to demonstrate the common descent of all animals. Today, thanks to evo devo, it is a given fact underpinned by an increasingly broad comparative approach that makes use of phylogenetic trees, gene expression profiling, and other modern tools. Evo devo has produced many extraordinary insights. Perhaps the most unexpected insight is exemplified by the fact that not a single biologist ever anticipated that the same genes controlling the design of an insect's body and organs would also control the design of our own bodies. Indeed, Carroll cites Ernst Mayr as having predicted that “the search for homologous genes is quite futile except in very close relatives.” We now not only know much about how complexity is constructed from a single cell into a whole animal, but also fully appreciate how its foundation in an ancient genetic toolkit provides irrefutable evidence of the common descent of all animals. In addition, to me, it is exhilarating that genes crucial in specifying early embryonic and limb development in arthropods and other animals have been co-opted to tinker with the formation of butterfly wing patterns in the final throws of development [3].

In comparison to Carroll's earlier book [2], *Endless Forms Most Beautiful* is a much more personal account of how the field has become established, its major achievements to date, and where it is heading. For any biologist or scientist interested in learning more about the principles of development and the evolution of diversity in animal form, this is the book to read; for any evo devo researcher, this is a fascinating tale of how one of the leading figures in the field views its contributions to science and its future directions.

Carroll also reasons persuasively that evo devo provides wonderful opportunities for grabbing the interest of a new generation of biologists. His account illustrates how research in evo devo is fun as well as challenging. As he puts it, “Biology without evolution is like physics without gravity.” Evolution of form is change in development, and with the new insights, this process provides a route for inspirational teaching: there seems little doubt that, for many in the classroom, this will prove more attention-grabbing than a population-genetics approach based on the idea that “evolution is change in gene frequency.” Perhaps of no less importance than the up-to-date nature of the field is the aesthetic beauty of many images of development, such as those that fill the two sections of color plates in this book. While biologists have always been enthralled by diagrams of adaptive radiations starring those “endless forms most beautiful,” I defy anyone not to find Carroll's images of the patterns of gene expression in development just as captivating, especially when one appreciates how they reveal the underlying principles.

Carroll is clearly a master storyteller. The ease with which he weaves his story will come as no surprise to anyone who has experienced one of his performances at the lectern. His easygoing prose is matched by his infectious enthusiasm and open curiosity for all levels of diversity in biology. Although Carroll comes from a developmental biologist's background, he is well-read in evolutionary biology, including the history of the field. This book is characterized by phrases that provide evocative illustrations of how the evolution of form occurs. One doesn't usually come across words like “stunning” and “eureka” in the standard textbook accounts of a new field, but their usage adds to the effectiveness of this book in capturing the spirit of the new discoveries. Metaphors are used in a most effective way; for example, the evolution of forks or paper clips within recent human history illustrates how novelties are followed by elaboration and modification to yield a diversification of form and functional roles in different ecological environments. Scientific jargon is kept to an absolute minimum. These rhetorical devices all add to the excitement of the account, and make the book accessible to scientist or layman.

*Endless Forms Most Beautiful* can also fulfill an important role in the public outreach of our science. If I needed a single book to convince a skeptic or an advocate of intelligent design that evo devo is an exciting and influential field in modern biology, and that the principle of common descent is not theory but fact, I would have to look no further. As Carroll himself says, the discoveries of evo devo are illuminating the evolutionary process and particular evolutionary events in powerful new ways. There is fierce debate centered on evolution versus intelligent design in several countries, most prominently in the United States but also in the Netherlands, where I am based—this book would provide an invaluable primer for anyone uncertain about the rigor of the basis for evolution. Carroll makes it clear how complexity evolves, using examples that lend clarity to the account. The fine details may not yet be all there, but many of the underlying principles have been made much more assessable and robust by the advances of evo devo. One optimistic message of Carroll's book is that a complete understanding of the evolution of many large-scale and complex changes in animal design is within our grasp. Likewise, work in evo devo will also reveal precisely how changes evolve in the details of animal design across related species.

Whilst the scope of his book is very broad, Carroll uses a straightforward progression of topics to support his ideas. The first half of the book presents a primer on the principles of embryonic development. The remarkable discoveries of developmental biology, of how fruit fly genetics led to some of the profound insights of evo devo, come alive in Carroll's words. I can still recall the sheer frisson of excitement generated by waves of new discoveries at the first developmental biology meetings that I attended 15 years ago. Specific details and complexities, as well as epigenetic phenomena, may be glossed over in the text, but the reader interested in delving more deeply can use the thorough concluding section on scientific sources. Also, while on first reading, phrases such as “multiple genetic switches” may jar the reader, they end up being excellent choices in terms of explaining the basic principles to a very broad audience. There are also intriguing mysteries—why does it take as long to a make a 32-cell human embryo as it does to make a complete tadpole?

In the second half of his book, Carroll expands on four ideas about animal development: (1) the modularity of animal architecture, (2) the genetic toolkit for building animals, (3) the geography of the embryo in space and time, and (4) that genetic switches determine the coordinates of toolkit gene action in the embryo. All this is covered with broad references to the fossil record, the Cambrian explosion, and the opportunities arising from new ecological environments and innovations in animal form. Using numerous examples, often from evo devo work coming out of his own laboratory, he then shows how animal forms evolve through changes in the geography of the embryo, and that evolution of form is largely about “teaching very old genes new tricks.” For example, his team found that a primitive Australian onychophoran, or velvet worm, has no fewer Hox genes than flies and other arthropods [4]. Thus, the modularity and dramatic diversification of arthropod limbs and their parts has not evolved through increasing the toolkit of Hox genes but rather through evolution of the regulatory machinery.

Carroll argues that the arena of evolutionary action is, indeed, largely within the rich operating instructions for the toolkit genes, an area of special, current interest in his own laboratory [5]. Form evolves largely by elaborating on, and tinkering with, how these genes are regulated through batteries of multiple switches. The forms of animal parts are the products of large numbers of switches and proteins that organize patterns in time and space; the same genes and associated signaling pathways are used again and again at different times in development and in different tissues. Research is beginning to bring this to life, although it will still be a long time before all the exquisite details are revealed for particular toolkit genes and structures across groups of related animal species. Carroll makes some predictions for the future in terms of the complexity of the logic that underlies evolution of form. He shows how even the most complex of adaptations arise in this way, with multifunctionality and redundancy providing the ecological background to the evolution of specialization in specific environments. The remodeling of limbs to produce new forms and functions, whether in arthropods or in vertebrates, is a matter of changing the geography of limb development in terms of the patterns of expression of toolkit genes in time and space.

In a section near the end of the book, Carroll begins to apply the framework of evo devo to the evolution of humans, including the human mind. He discusses how the discoveries and perspectives of evo devo have already enriched and expanded on the foundations of evolutionary thinking—there is, however, clearly much more to come. He concludes with an eloquent plea that understanding the history of this planet is the key to our successful stewardship of it. I hope that he is right.

*Endless Forms Most Beautiful* reinforces the prediction that the foreseeable future will see such advances in evo devo that we will know in wonderful detail how both major and much more subtle changes in animal form are generated, and how the underlying developmental processes have evolved. One remaining challenge will be to match such a sophisticated understanding of how form and variation in form are generated with comparable detail from the perspectives of function and fitness in ecological environments. Another challenge will be to compare the properties of evolution in form and morphology with how life histories, behaviors, and other components of the whole functional phenotype of organisms evolve—it will be fascinating to see the results of an evo devo type of framework applied with equal force to these other facets of the phenotype. Finally, it remains to be seen how the processes of development themselves bias or channel evolution, such that even the most vivid examples of adaptive radiation are shaped not only by natural selection but also by how development works and is regulated.
